# Individual action, sharing scarce resources, sharing information? A study on how to effectively manage forest pests and diseases based on carbon trading

**DOI:** 10.1371/journal.pone.0322237

**Published:** 2025-04-28

**Authors:** Shansong Wu, Yuntao Bai, Jiahao Li, Yueling Yang

**Affiliations:** 1 School of Accounting, Wuxi Taihu University, Wuxi, China; 2 Business School, Shandong Management University, Jinan, China; 3 Information Engineering School, Shandong Management University, Jinan, China; 4 Department of Economics and Rural Development, Gembloux Agro-Bio Tech, University of Liège, Gembloux, Belgium; Universitas Airlangga, INDONESIA

## Abstract

In recent years, forest pests and diseases have had a significant impact on forest ecosystems. To incentivize corporations to manage forest pests and diseases, the government provides certain carbon compensations to enterprises involved in this management. In the process of controlling forest pests and diseases, the modes of collaboration between the government and corporations are primarily categorized into three modes: independent action, scarce resource sharing, and information sharing. To determine the applicability of each relational mode, this paper constructs three differential game models and compares and analyzes the equilibrium results obtained from these modes. The research indicates that if the cost of government-managed forest pest and disease control is high and the benefits of such control are low, then the scarce resource sharing mode can offer the government the maximum benefit; conversely, the information sharing mode can provide the government with the greatest benefit. If the cost and benefits of corporate-managed forest pest and disease control are low, then the information sharing mode can offer corporations the maximum benefit; otherwise, the scarce resource sharing mode can provide corporations with the greatest benefit.

## 1. Introduction

The severity of forest pests and diseases has profound implications for ecosystems, economies, and human livelihoods. Forests cover a vast majority of the Earth’s land area and are crucial for maintaining biodiversity, purifying air and water, controlling climate, protecting soil, and providing numerous goods and services to humanity.

Here are some of the issues and consequences that can arise from serious forest pest and disease outbreaks. Firstly, ecosystem damage. Pests and diseases can reduce plant populations, affecting forest structure and function. This may further degrade habitats, negatively impact wildlife, and diminish biodiversity.

Secondly, reduced carbon sequestration. Forests are major carbon sinks, absorbing carbon dioxide through photosynthesis. Pests and diseases leading to tree mortality reduce the carbon storage capacity of forests and can result in the release of carbon back into the atmosphere, exacerbating global climate change.

Thirdly, economic losses. Forests offer immense economic value, including timber, pulp, and other forest products. Epidemics of pests and diseases can reduce timber yields, leading to economic losses and affecting communities and industries dependent on forests.

Fourthly, increased risk of forest fires. Plants weakened or killed by pests and diseases are more susceptible to burning, raising the risk of forest fires. More intense fires can further lower carbon capture capabilities and lead to more greenhouse gas emissions.

Fifthly, threats to public safety. Dead or infected trees may fall, posing direct hazards to human activity areas such as roads, residential zones, and public utilities. Forest pests and diseases are among the primary factors affecting forest health and productivity, caused by a variety of reasons including biological factors, environmental factors, and human activities.

These factors interact and collectively impact forest health and the stability of ecosystems. Therefore, effective forest pest and disease management strategies need to consider these factors comprehensively, adopting diversified prevention and control measures.

Given the potential risks of forest pests and diseases to ecosystems, economies, and public health, effective management of these threats is essential. Strategies for managing forest pathogens and pests could include the following. First, monitoring and early warning. Establishing and maintaining a forest health monitoring system at various levels is a critical first step. By integrating remote sensing technology with ground patrols and sampling, signs of pests and diseases can be detected and responded to promptly.

Second, integrated management. Adopting Integrated Pest Management (IPM) strategies that combine physical, chemical, biological, and cultural methods of pest control is vital. This includes using natural enemies and biopesticides to control pest populations, as well as altering forest management practices to reduce the impact of pests and diseases.

Third, resistance breeding. By selecting and breeding, developing tree species and varieties that are resistant to specific pests and diseases can help reduce dependence on chemical pesticides.

Fourth, enhancing forest diversity. Promoting the planting of diverse species combinations can reduce the outbreaks and spread of pests and diseases. Diverse plant communities are often more resistant to pests and diseases.

Fifth, restoration and reforestation. Forest areas severely damaged by pests and diseases require ecological restoration, including reforestation and natural regeneration, to restore the structure and function of ecosystems.

Managing forest pests and diseases is a long-term and complex process that requires continual adaptation to environmental changes and emerging challenges. Effective management strategies rely on joint efforts at the global, regional, and local levels, and on adopting scientific, sustainable, and socio-economically viable management practices.

## 2. Literature review

### 2.1. Previous research

As research into forest pest and disease management continues to advance, a significant body of work has emerged. Some scholars have analyzed the impact of physical and technical measures on the control of these forest afflictions. For example, Hagge et al. [1] assessed the effects of mechanical pest control strategies in managing pest and disease issues in coniferous forests. Fang et al. [2] explored how designing helicopter routes could improve the management of forest pests and diseases. Xin and Wang [3] investigated the use of deep learning for image recognition of forest pests and diseases. Additionally, León-Bañuelos et al. [4] suggested that the presence of harmful organisms and parasitic plants in forests could be analyzed using unmanned aerial vehicles and algorithms. These studies encompass physical and technical approaches such as machinery, route design, image recognition, and algorithms for the management of forest pests and diseases.

Some scholars have analyzed the impact of biological and ecological approaches on the management of forest pests and diseases. For instance, Aristizábal and Metzger [5] studied the effect of spatial layout on the control of forest pests and diseases. Yang et al. [6] researched meteorological enemies of pests, highlighting the significant role weather conditions play in the management of forestry pests. Qin [7] suggested that establishing a platform for correlating meteorological conditions with the dynamics of harmful organisms in forestry would enhance the forecasting and early warning of harmful organisms. Ihsan and Sumarmin [8] examined the impact of birds on pest management in oil palm plantations. Grace et al. [9] investigated the use of bacteriophages for the control of forest pests and diseases. These studies encompass biological or ecological methods such as spatial arrangement, natural enemies, meteorological early warning, and bacteriophages for the prevention and control of forest pests and diseases.

Some scholars have examined the impact of policy and economic incentives on forest pest and disease management. For instance, Sheremet et al. [10] investigated how to implement policies that motivate private forest owners to engage in pest and disease control efforts. Lovett et al. [11] argued for the necessity of controlling the distribution channels of wood products. Garnas et al. [12] contended that cost sharing, the growth and maintenance of resources and capabilities, and a more comprehensive research program are crucial for the long-term success of biological control. These studies encompass policy and economic incentives such as motivational measures, distribution channels, cost-sharing, and resource-sharing for the management of forest pests and diseases.

The aforementioned studies primarily elucidate approaches to managing forest pests and diseases through physical, technical, biological, ecological, policy, and economic incentive methods. Forests, as significant carbon sinks, absorb and sequester atmospheric carbon dioxide through photosynthesis, playing a crucial role in mitigating climate change. However, the outbreak of pests and diseases can compromise forest health, leading to reduced tree growth, mortality, and even large-scale degradation, which not only diminishes the carbon sequestration capacity of forests but may also result in the re-release of stored carbon into the atmosphere, exacerbating the greenhouse effect. Scientific management of forest pests and diseases is fundamental to maintaining the carbon sink function of forests and is essential for ensuring that forests play a positive role in the carbon cycle. Therefore, some scholars have conducted research on forest carbon sinks. For example, the reduction of Siberian carbon sinks due to forest disturbances [13]; the implications of retained ammonium and nitrate deposition for global forest carbon sinks [14]; regrown tropical forests as substantial carbon sinks [15]; a global synthesis assessment of the carbon potential of natural forests [16]; and the estimation of carbon balance recovery time following forest logging [17]. These scholars discuss forest carbon sinks from the perspectives of their impact on carbon sequestration, the assessment of forest carbon sinks, and their restoration. As a significant source of carbon credits, the carbon absorption capacity of forest carbon sinks directly influences the supply and value of the carbon trading market. By effectively managing pests and diseases, the functionality of forest carbon sinks can be preserved and enhanced, thereby providing the carbon trading market with a greater quantity of high-quality carbon credits and fostering the development of a low-carbon economy.

### 2.2. Deficiency of previous research

However, the aforementioned studies still exhibit some shortcomings. Firstly, there is a lack of specific mechanisms for integrating carbon trading with pest and disease management. Despite exploring multiple aspects of forest carbon sequestration and pest and disease prevention, there is a scarcity of research on specifically integrating carbon trading mechanisms into forest pest and disease management, such as incentivizing forest owners to adopt more effective pest and disease management strategies through carbon credits.

Secondly, there is a lack of interdisciplinary approaches to tackle complex issues. Research tends to focus on singular domains, such as mechanical control, ecological methods, or policy measures, without adequately considering how to effectively integrate ecology, economics, technology, and social policy to collectively enhance forest pest and disease management based on carbon trading.

Thirdly, there is an insufficient study on the management and optimal utilization of scarce resources. An analysis of how to best utilize limited resources, such as funding and manpower, for effective pest and disease management within the broader context of integrating carbon trading and forest carbon sequestration is lacking.

### 2.3. Contribution and significance

Facing these deficiencies, this paper introduces the following innovations and contributions. First, it proposes an innovative mode that integrates carbon trading with forest pest and disease management. This mode, which has been developed and tested, combines forest pest and disease management with carbon trading to motivate forest owners and managers to adopt eco-friendly and carbon-efficient practices. The mode includes economic incentives, technical support, and a policy framework to ensure long-term and sustainable forest health and its carbon sequestration capacity. Second, the application of interdisciplinary approaches. An integrated study involving ecology, information technology, economics, and social science explores how carbon trading can enhance the efficiency of forest pest and disease prevention and control. Third, the optimization of resource management and usage. The paper researches and implements a resource optimization allocation mode to ensure the effective use of limited resources in pest and disease management. This involves optimizing the distribution of manpower and financial support, while maximizing the carbon sequestration potential of forests.

In this article, the three modes of individual action, sharing scarce resources and sharing information are described first, and then the differential game model under these three modes is established. The HJB equation is used to solve the differential game model, and the equilibrium solutions under different modes are compared and analyzed. Finally, the research conclusion is drawn.

The research presented in this article is of significant importance, primarily manifested in the following aspects. First, it promotes forest health and addresses climate change. Forests, as crucial carbon sinks on Earth, have their health directly linked to the mitigation of and adaptation to global climate change. Effective management of forest pests and diseases not only ensures the health and stability of forest ecosystems but also enhances their capacity to absorb carbon dioxide, thereby combating climate change. Second, it promotes the sustainable management of forest resources. By studying pest and disease management methods based on carbon trading, new perspectives and tools can be offered for the sustainable management of forest resources. Incentivizing individual actions, effectively utilizing scarce resources, and sharing information can improve management efficiency and promote the sustainable use of forest resources. Third, it innovates economic incentive mechanisms. Introducing carbon trading into forest pest and disease management explores new economic incentives, encouraging more private and public forest landowners to engage in forest conservation and pest control. These economic incentives can integrate market forces to promote a win-win scenario for ecological protection and economic development.

## 3. Methodology

### 3.1. Problem description, variable definition, and hypothesis

#### 3.1.1. Problem description.

From the perspective of game theory, the interaction between governments and corporations can be viewed as a strategic interaction process, where the management of forest pests and diseases becomes a common concern under carbon trading scenarios. In this game, although the goals of the participants may vary, they need to collaborate to achieve the common goal of carbon emission reduction while addressing forest pests and diseases. The government typically plays the role of policy-maker and regulator in the carbon trading market, setting emission reduction targets, regulating the carbon market, and possibly providing financial incentives or compensation for forest pest and disease management. The government’s optimization strategies are as follows: First, setting clear rules and incentives. The government needs to establish an effective policy framework and incentive mechanisms to encourage corporations to participate in carbon trading through forest protection and pest and disease management. Second, regulation and verification. Ensuring the authenticity and transparency of forest carbon sink projects through appropriate regulation and third-party verification. Third, subsidies and support. Providing financial support for actions taken to manage forest pests and diseases, such as tax reductions or direct subsidies [10]. Corporations participate in the carbon trading market, potentially reducing emissions directly or investing in forest carbon sink projects to offset emissions, with forest health management being part of emission reduction projects. The corporations’ optimization strategies include: First, cost-benefit analysis. Selecting projects that both effectively manage forest pests and diseases and bring emission reduction certificates to the corporation. Second, long-term planning. Considering the long-term carbon storage capacity and sustainability of forest health when investing in forest pest and disease management. Third, social responsibility. Corporate participation in forest management can also serve as a reflection of Corporate Social Responsibility (CSR), enhancing brand value. In such a gaming field, the government needs to develop strong policies and incentives to ensure corporations see the economic benefits of investing in forest pest and disease management, making it an attractive operation. Meanwhile, corporations seek investment projects with high cost-effectiveness and controllable risk. Therefore, the focus of the game is to find a balance point acceptable to both parties. To achieve this, the parties may take the following approaches: First, cooperative strategies. Governments and corporations collaborate through contracts, agreements, or joint projects to advance forest pest and disease management. Second, negotiation and standard setting. Jointly determining the qualification standards and verification methods for forest carbon sink projects participating in carbon trading, as well as how to assess the effectiveness of forest pest and disease management. Third, dynamic adjustments. As market conditions change and forest pest and disease management technology advances, governments and corporations need to continuously adjust their strategies and policies. Ultimately, through this gaming process, both parties aim to achieve a win-win state: the government achieves its goals of carbon emission reduction and forest protection, while corporations obtain the carbon emission reduction certificates needed in the carbon market and enhance their environmental responsibility image. Achieving this goal requires foresighted policy-making, proactive execution by corporations, and continuous communication and adaptation between both parties.

The process of forest pest and disease management based on carbon trading between governments and corporations is long-term, dynamic, and continuous, influenced by numerous variables and perpetually changing environmental factors. These factors include the complexity of forest ecosystems, climate change, the evolution of economic policies, and technological advances. Firstly, the complexity of forest ecosystems entails that forest ecosystems are highly complex and variable, with the dynamics of pests and diseases often being difficult to predict and control. Effective management requires sustained monitoring, research, and adaptive management measures, necessitating long-term commitment and resource investment. Secondly, climate change exerts profound influences on forest pests and diseases, potentially altering their populations and distributions, mandating continual adjustments to measures to accommodate these changes. Thirdly, economic and policy developments, including the fluctuation of global and regional economic policies such as developments in the carbon market, carbon pricing, and related incentive mechanisms, impact the decision-making and conduct of both governments and enterprises. These policies and market conditions are dynamic in themselves, necessitating real-time strategic adjustments to adapt to new economic contexts. Fourthly, technological progress can improve the efficiency and cost-effectiveness of forest pest and disease management, requiring governments and corporations to continuously update their management strategies to leverage these technological advancements. Fifthly, random events and uncertainties frequently characterize pest and disease outbreaks often triggered by uncontrollable natural factors, such as extreme weather events, and markets and policies may also be affected by unpredictable incidents, such as political shifts or financial crises. Therefore, governments and corporations must incorporate this uncertainty into their management strategies to respond flexibly. Lastly, the accumulation of scientific knowledge is an ongoing process. As scientific research progresses, our understanding of forest pests and diseases continually improves. Policies and management measures require constant updates to reflect the latest scientific discoveries. For these reasons, the process of forest pest and disease management is inevitably continuous, dynamic, and necessitates long-term commitment and strategic planning. The collaborative capabilities and adaptability of governments and corporations, as well as their responsiveness to the ever-changing environmental conditions, will play an essential role in this process.

In the forest carbon emission trading system, governments and enterprises can jointly participate in the management of forest pests and diseases through individual action mode, scarce resource sharing mode, and information sharing mode. Under the individual action mode, the government promotes forest health and ensures the fairness and transparency of carbon trading through legislative, policy formulation, and financial incentives, such as setting forest management standards, emission reduction targets, and market regulation mechanisms, providing research funding and infrastructure construction support, and strengthening public education and professional personnel training [18]. Enterprises, on the other hand, enhance forest health and improve carbon sink capacity by directly investing in management projects, purchasing carbon credits, managing supply chains, and engaging in sustainable forest management. Although these actions are described as independent, effective cooperation and coordination are crucial for achieving management objectives. The scarce resource sharing mode emphasizes improving resource utilization efficiency, reducing costs, promoting technological innovation, and minimizing project risks through public-private partnerships (PPP), joint investments, technology and knowledge sharing, market information flow, joint research and development, and incentive measures [9]. The information sharing mode advocates for the establishment of information platforms, implementation of data sharing and transparency requirements, government organization of monitoring and reporting systems, active participation of enterprises and provision of data support, and joint funding of research activities, all of which help optimize forest management strategies, provide key information for decision-making to market participants, and ensure the fairness, efficiency, and environmental benefits of the carbon trading market [7]. These three modes complement each other and together promote the effective management of forest pests and diseases and the reduction of carbon emissions.

Forest pest and disease management can adopt various modes, including individual action, scarce resource sharing, and information sharing. Each mode has its own advantages and disadvantages, and depending on different circumstances and conditions, different management strategies may be necessary. Below is a comparative analysis of these three modes. The advantages of the individual action mode are mainly reflected in the following aspects: First, decision-making speed. Individual actors do not need to consult with others and can respond quickly to pest and disease threats. Second, complete control. Each actor has full control over the measures they take. Third, customized solutions. Each actor can develop specialized pest and disease management strategies based on their own situation and resources. The disadvantages of individual action mainly include: First, inefficiency. Each actor may lack sufficient resources and expertise to effectively address the problem. Second, lack of coordination. Independent actions may lead to a lack of consistency in overall management strategies, reducing the effectiveness of pest and disease management as a whole. Third, resource limitations. Individual actors may lack sufficient funds and technology to implement effective management measures. The advantages of the scarce resource sharing mode are mainly reflected in the following aspects: First, cost-effectiveness. By sharing resources, participants can reduce the costs for individual actors. Second, resource integration. It allows for the pooling of limited resources to more powerfully address larger-scale or more complex pest and disease issues. Third, enhanced collaboration. Sharing resources promotes the exchange of information and experience, enhancing the efficiency of resource use. The disadvantages of scarce resource sharing mainly include: First, coordination challenges. Resource sharing requires complex coordination work and may become deadlocked due to differing needs or conflicts of interest. Second, unequal contributions. Parties involved in resource sharing may disagree on the amount of contributions, potentially leading to unequal investments. The advantages of the information sharing mode are mainly reflected in the following aspects: First, enhanced knowledge base. Sharing information can help parties learn from others’ experiences and improve problem-solving capabilities. Second, risk warning. Timely sharing of pest and disease dynamics can serve as an early warning system, reducing the impact of pests and diseases. Third, lower cost. Compared to resource sharing, information sharing generally costs less and is easier to implement. The disadvantages of information sharing mainly include: First, information quality. The quality of shared information may be uneven, not necessarily usable by everyone. Second, data privacy. Some data might involve sensitive information, requiring data privacy and security to be ensured during sharing. Third, implementation gap. Even with proper information sharing, it is necessary to ensure that participants have the ability to translate knowledge into practice. Generally speaking, by integrating these three modes and formulating a coordinated, resource-integrated, and transparent comprehensive management strategy based on actual situations, the best effect in managing forest pests and diseases might be achieved.

**Fig 1:**
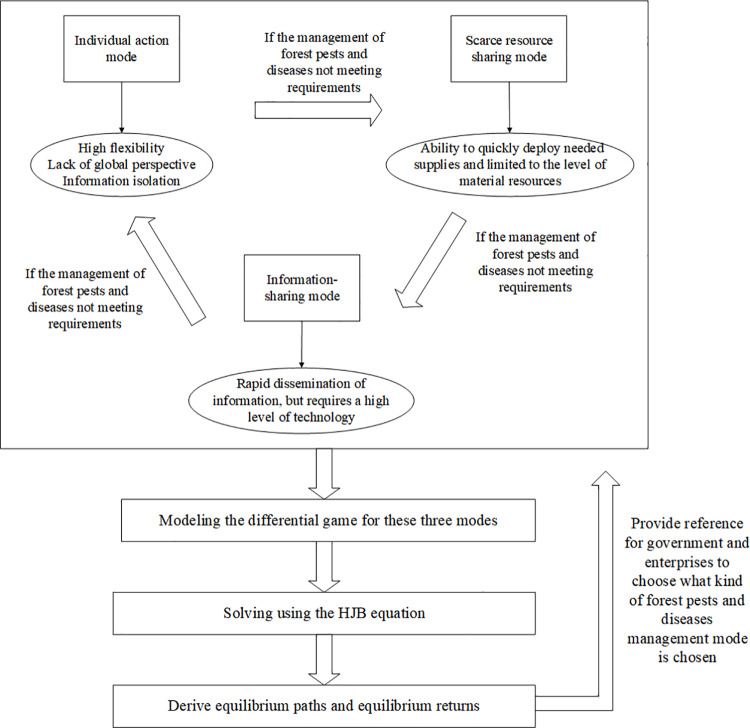
Mechanism flow.

In order to describe the mechanism flow of this paper more clearly, Fig 1 is made in this paper.

#### 3.1.2. Variable definition.

When constructing the differential game model in this article, many parameters and variables are designed. These parameters and variables are defined as shown in Table 1_._

**Table 1:** The main definition of variables and parameters in this article.

Variables and parameters	Specific meaning
*Y=*{*A,S,I*}	three modes for the management of forest pests and diseases (individual action, sharing of scarce resources, sharing of information)
independent variable	
*F*_*Y*1_(*t*)	the extent of the government’s efforts to manage forest pests and diseases under mode *Y*
*F*_*Y*2_(*t*)	he extent of the enterprises’ efforts to manage forest pests and diseases under mode *Y*
*x*_*Y*1_(*t*)	the government’s reputation for managing forest pests and diseases under mode *Y*
*x*_*Y*2_(*t*)	the enterprises’ reputation for managing forest pests and diseases under mode *Y*
parameter	
*ρ*	the discount rate that occurs over time, 0≤*ρ*≤1
*δ*	decay of reputation, *δ*>0
*b*_1_, *b*_2_	gains from the government or enterprises management of forest pests and diseases at a unit level, *b*_1_, *b*_2_>0
*c*_1_,*c*_2_	costs to the government or enterprises of managing forest pests and diseases at a unit level, *c*_1_, *c*_2_>0
*C*_*o*_	government carbon offsets to enterprises, *C*_*o*_>0
*a*_1_,*a*_2_	the reputation gained by government or enterprises for managing forest pests and diseases at a unit level, *a*_1_,*a*_2_>0
*c*_*S*_	cost of government sharing of scarce resources, *c*_*S*_>0
*β*_S_	extent to which scarce resources help firms manage forest pests and diseases, *β*_S_>0
*a*_*S*_	reputation for the government due to sharing of scarce resources, *a*_*S*_>0
*l*	the positive effects of reputation, *l*>0
*b*_*I*_	increased benefits of forest pest and disease management from information sharing, *b*_*I*_>0
*C*_*I*_	costs of information sharing, *C*_*I*_>0
function	
*J*_*Y*1_(*t*)	the social welfare function of government under the management mode *Y*
*J*_*Y*2_(*t*)	the social welfare function of enterprises under the management mode *Y*
*V*_*Y*1_(*t*)	the social benefits of government under the management mode *Y*
*V*_*Y*2_(*t*)	the social benefits of enterprises under the management mode *Y*

#### 3.1.3. Hypothesis.

Hypothesis 1: Scarce resource sharing can cost the government extra as well as gain extra prestige.

Engaging in forest pest and disease management through carbon trading represents a market-based mechanism in the field of environmental economics for governments and corporations. In this process, governments and corporations may need to share some scarce resources, such as expertise, technology, or monitoring equipment, to ensure the health of forest ecosystems and effective pest and disease management. Sharing scarce resources could lead governments to incur additional costs *c*_*S*_ but also gain additional reputation *a*_*S*_.

The reasons for the additional costs *c*_*S*_ are as follows. First, specialized technologies or services can be expensive, especially if these resources are highly specialized or need to be imported from abroad. Second, sharing scarce resources may require additional logistics, coordination, and management expenses, which require time and financial investment. Third, governments may need to pay corporations for the use of licenses, royalties, or other forms of compensation, especially if these resources are accessed through patented technology or proprietary knowledge.

Meanwhile, the reasons for the extra reputation *a*_*S*_ are as follows. First, by collaborating with corporations and investing in environmental management actions, governments demonstrate their commitment to sustainable forest management and addressing climate change, which increases public trust in government environmental policies and actions. Second, successful pest and disease management and forest restoration can improve the ecological health of regions or even globally, thereby enhancing the government’s international reputation in environmental protection. Third, participating in carbon trading projects can bring governments international recognition, such as carbon credits obtained through mechanisms of the United Nations Framework Convention on Climate Change (UNFCCC).

To ensure the cooperation between governments and corporations is genuinely effective and sustainable, it is necessary for governments to carefully analyze the potential costs and benefits of participating in carbon trading projects. Moreover, it is essential to report the progress and carbon reduction effects of projects openly and transparently, ensuring the public is aware of their efforts and achievements. This can enhance their reputation in environmental governance while maintaining fiscal responsibility.

Hypothesis 2: Scarce resource sharing can lead to an increase in enterprises’ earnings *b*_2_.

In the context of forest pest and disease management through carbon trading, the sharing of scarce resources among governments and corporations can increase enterprises’ earnings *b*_2_ for several reasons. First, cost reduction. By sharing resources with governments, corporations can lower the costs of investing in forest management and administration on their own. For instance, corporations might not need to purchase expensive management equipment or establish a complete monitoring system independently, as they can utilize the existing resources and capabilities of the government. Eliminating redundant investments can reduce the financial burden on corporations and enhance their overall profitability. Second, enhanced efficiency. Sharing resources contributes to improved work efficiency. The integration of professional technical and human resources helps projects operate efficiently, meaning corporations can achieve forest management objectives more rapidly and enhance their competitiveness in the market due to quick responsiveness. Third, increased project success rates. Sharing resources means pooling the strengths of all parties involved, increasing the likelihood of successful pest and disease management. Successful forest management benefits the maintenance of the forest assets invested by the corporation, ensuring the long-term viability and profitability of carbon sequestration projects. By collaborating with governments in sharing scarce resources and jointly participating in the carbon market, corporations can effectively leverage their respective advantages, reducing the risks and costs faced by each party and increasing the return on business investments. In the context of globally increasing pressure to reduce carbon emissions, this collaborative mode is particularly advantageous for corporations.

Hypothesis 3: Information sharing can lead to more efficient governance for both government and enterprises.

In the collaborative process of forest pest and disease management through carbon trading between governments and corporations, the role of information sharing and its benefits *b*_*I*_ in enhancing management efficiency can be understood from several perspectives. First, improving decision-making quality. With more data and information being shared, both governments and corporations can gain a more comprehensive understanding and insights, aiding them in making wiser and more effective management decisions. This includes knowledge about the types, distribution, impacts, and transmission pathways of forest pests and diseases. Second, reducing duplication of work. If both parties collect and analyze information without sharing, it would lead to duplication of efforts and a waste of resources. Information sharing helps ensure efforts are not duplicated, thus focusing resources and attention on other critical aspects of pest and disease management. Third, enabling rapid response. An information-sharing mechanism ensures participants quickly access the latest information on pest and disease outbreaks. Consequently, governments and corporations can respond swiftly to emerging threats with emergency measures to avoid or minimize large-scale outbreaks. Overall, information sharing allows governments and corporations to break down information silos and better collaborate, achieving efficient and effective pest and disease management strategies, which is crucial for enhancing management efficiency and successfully managing forest health.

**Fig 2:**
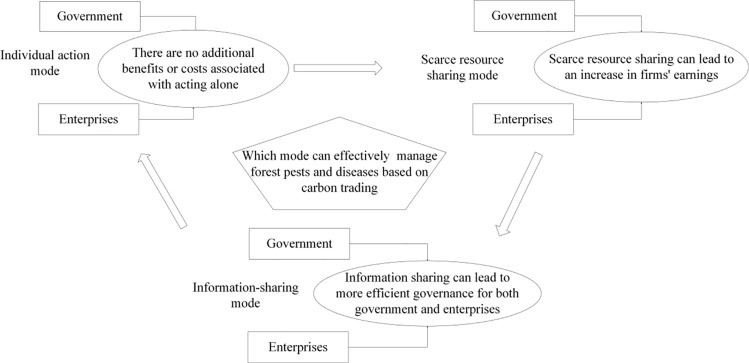
Relationship between three modes of forest pests and diseases management.

The relationship between the three modes of forest pests and diseases management is shown in Fig 2.

If the government and corporations manage forest pests and diseases under the individual action mode, their social welfare function can be expressed as follows:


$$J_{A1}=\int_{0}^{\infty } [b_{1}F_{A1}\left(t\right)-\frac{c_{1}}{2}F_{A1}^{2}\left(t\right)-C_{O}\left.+lx_{A1}\left(t\right)\right]e^{-\rho t}dt$$ (1)

$$J_{A2}=\int_{0}^{\infty } [b_{2}F_{A2}\left(t\right)-\frac{c_{2}}{2}F_{A2}^{2}\left(t\right)+C_{O}\left.+lx_{A2}\left(t\right)\right]e^{-\rho t}dt$$ (2)

In the above formula, $b_{1}F_{A1}\left(t\right)$ refers to the benefits government gains from managing forest pests and diseases under a solitary action mode. $\frac{c_{1}}{2}F_{A1}^{2}\left(t\right)$ reflects the costs incurred by the government for managing forest pests and diseases under a solitary action mode. $lx_{A1}\left(t\right)$ signifies the long-term benefits that reputation brings to the government under a solitary action mode. $b_{2}F_{A2}\left(t\right)$ denotes the benefits corporations gain from managing forest pests and diseases under a solitary action mode. $\frac{c_{2}}{2}F_{A2}^{2}\left(t\right)$ represents the costs incurred by corporations for managing forest pests and diseases under a solitary action mode. $lx_{A2}\left(t\right)$ indicates the long-term benefits that reputation brings to corporations under a solitary action mode.

The change in the reputation of government and corporations under the individual action mode can be expressed as:


$${\dot{x}}_{A1}\left(t\right)=a_{1}F_{A1}\left(t\right)-\delta x_{A1}\left(t\right)$$ (3)

$${\dot{x}}_{A2}\left(t\right)=a_{2}F_{A2}\left(t\right)-\delta x_{A2}\left(t\right)$$ (4)

$a_{1}F_{A1}\left(t\right)$ signifies the reputation gained by the government in managing forest pests and diseases under a solitary action mode. $a_{2}F_{A2}\left(t\right)$ denotes the reputation earned by corporations in managing forest pests and diseases under a solitary action mode. $\delta x_{A1}\left(t\right)$ represents the deterioration of government reputation under a solitary action mode. $\delta x_{A2}\left(t\right)$ indicates the decline in corporate reputation under a solitary action mode.

If the government and corporations manage forest pests and diseases under the sharing scarce resources mode, their social welfare function can be expressed as follows:


$$J_{S1}=\int_{0}^{\infty } [b_{1}F_{S1}\left(t\right)-\frac{c_{1}+c_{S}}{2}F_{S1}^{2}\left(t\right)-C_{O}\left.+lx_{S1}\left(t\right)\right]e^{-\rho t}dt$$ (5)

$$J_{S2}=\int_{0}^{\infty } [b_{2}\ln\left(\text{e}+\beta_{S}\right)F_{S2}\left(t\right)-\frac{c_{2}}{2}F_{S2}^{2}\left(t\right)+C_{O}\left.+lx_{S2}\left(t\right)\right]e^{-\rho t}dt$$ (6)

In the above formula, $b_{1}F_{S1}\left(t\right)$ represents the benefits the government obtains from managing forest pests and diseases under a scarce resource sharing mode. $\frac{c_{1}+c_{S}}{2}F_{S1}^{2}\left(t\right)$ reflects the costs incurred by the government in managing forest pests and diseases under a scarce resource sharing mode. $\frac{c_{S}}{2}F_{S1}^{2}\left(t\right)$ denotes the cost of sharing scarce resources for the government under a scarce resource sharing mode. $lx_{S1}\left(t\right)$ indicates the long-term benefits that the government derives from its reputation under a scarce resource sharing mode. $b_{2}\ln\left(\text{e}+\beta_{S}\right)F_{S2}\left(t\right)$ signifies the benefits corporations receive from managing forest pests and diseases under a scarce resource sharing mode. $\frac{c_{2}}{2}F_{S2}^{2}\left(t\right)$ represents the costs incurred by corporations in managing forest pests and diseases under a scarce resource sharing mode. $lx_{S2}\left(t\right)$ implies the long-term benefits that reputation yields for corporations under a scarce resource sharing mode.

The change in the reputation of government and corporations under the sharing scarce resources mode can be expressed as:


$${\dot{x}}_{S1}\left(t\right)=\left(a_{1}+a_{S}\right)F_{S1}\left(t\right)-\delta x_{S1}\left(t\right)$$ (7)

$${\dot{x}}_{S2}\left(t\right)=a_{2}F_{S2}\left(t\right)-\delta x_{S2}\left(t\right)$$ (8)

In the above formula, $a_{S}F_{S1}\left(t\right)$ signifies the reputation the government gains from sharing scarce resources under a scarce resource sharing mode. $a_{1}F_{S1}\left(t\right)$ denotes the reputation earned by the government in managing forest pests and diseases under a scarce resource sharing mode. $a_{2}F_{S2}\left(t\right)$ indicates the reputation corporations gain from managing forest pests and diseases under a scarce resource sharing mode. $\delta x_{S1}\left(t\right)$ reflects the decline in government reputation under a scarce resource sharing mode. $\delta x_{S2}\left(t\right)$ illustrates the deterioration of corporate reputation under a scarce resource sharing mode.

If the government and corporations manage forest pests and diseases under the sharing information mode, their social welfare function can be expressed as follows:


$$J_{I1}=\int_{0}^{\infty } [\left(b_{1}+b_{I}\right)F_{I1}\left(t\right)-\frac{c_{1}}{2}F_{I1}^{2}\left(t\right)-C_{I}-C_{O}\left.+lx_{I1}\left(t\right)\right]e^{-\rho t}dt$$ (9)

$$J_{I2}=\int_{0}^{\infty } [\left(b_{2}+b_{I}\right)F_{I2}\left(t\right)-\frac{c_{2}}{2}F_{I2}^{2}\left(t\right)-C_{I}+C_{O}\left.+lx_{I2}\left(t\right)\right]e^{-\rho t}dt$$ (10)

In the above formula, $\left(b_{1}+b_{I}\right)F_{I1}\left(t\right)$ reflects the benefits accrued to the government from managing forest pests and diseases under an information sharing mode. $\frac{c_{1}}{2}F_{I1}^{2}\left(t\right)$ encompasses the costs incurred by the government in the management of forest pests and diseases under an information sharing mode. $b_{I}F_{I1}\left(t\right)$ captures the enhancement of benefits in forest pest and disease management attributable to information sharing. $C_{I}$ denotes the cost of information sharing under an information sharing mode. $lx_{I1}\left(t\right)$ conveys the long-term benefits reputation provides to the government under an information sharing mode. $\left(b_{2}+b_{I}\right)F_{I2}\left(t\right)$ signifies the benefits enterprises gain from managing forest pests and diseases under an information sharing mode. $\frac{c_{2}}{2}F_{I2}^{2}\left(t\right)$ represents the costs borne by enterprises in managing forest pests and diseases under an information sharing mode. $lx_{I2}\left(t\right)$ implies the long-term benefits that reputation yields for enterprises under an information sharing mode.

The change in the reputation of government and corporations under the sharing information mode can be expressed as:


$${\dot{x}}_{I1}\left(t\right)=a_{1}F_{I1}\left(t\right)-\delta x_{I1}\left(t\right)$$ (11)

$${\dot{x}}_{I2}\left(t\right)=a_{2}F_{I2}\left(t\right)-\delta x_{I2}\left(t\right)$$ (12)

In the above formula, $a_{1}F_{I1}\left(t\right)$ signifies the reputation obtained by the government for managing forest pests and diseases under an information sharing mode. $a_{2}F_{I2}\left(t\right)$ denotes the reputation gained by enterprises in managing forest pests and diseases under an information sharing mode. $\delta x_{I1}\left(t\right)$ represents the decline in government reputation within the context of an information sharing mode. $\delta x_{I2}\left(t\right)$ illustrates the deterioration of corporate reputation under an information sharing mode.

## 4. Results

In the differential game, the government and corporations in the process of forest pests and diseases control are not only affected by control variables and parameters, but also change over time. In order to better calculate the control benefits and social benefits, the HJB formula is used. The HJB formula is a partial differential equation, which is the core of optimal control.

### 4.1. HJB formula

Under the individual action mode, the HJB equation of the social welfare function of the government and corporations are:


$$\rho V_{A1}=\underset{F_{A1}\left(t\right)}{\max} \{ [b_{1}F_{A1}\left(t\right)-\frac{c_{1}}{2}F_{A1}^{2}\left(t\right)-C_{O}+\left.lx_{A1}\left(t\right)\right]+\left.\frac{\partial V_{A1}}{\partial x_{A1}}\left[a_{1}F_{A1}\left(t\right)-\delta x_{A1}\left(t\right)\right]\right\}$$ (13)

$$\rho V_{A2}=\underset{F_{A2}\left(t\right)}{\max} \{ [b_{2}F_{A2}\left(t\right)-\frac{c_{2}}{2}F_{A2}^{2}\left(t\right)+C_{O}+\left.lx_{A2}\left(t\right)\right]+\left.\frac{\partial V_{A2}}{\partial x_{A2}}\left[a_{2}F_{A2}\left(t\right)-\delta x_{A2}\left(t\right)\right]\right\}$$ (14)

Under the sharing scarce resources mode, the HJB equation of the social welfare function of the government and corporations are:


$$\rho V_{S1}=\underset{F_{S1}\left(t\right)}{\max} \{ [b_{1}F_{S1}\left(t\right)-\frac{c_{1}+c_{S}}{2}F_{S1}^{2}\left(t\right)-C_{O}+\left.lx_{S1}\left(t\right)\right]+\left.\frac{\partial V_{S1}}{\partial x_{S1}}\left[\left(a_{1}+a_{S}\right)F_{S1}\left(t\right)-\delta x_{S1}\left(t\right)\right]\right\}$$ (15)

$$\rho V_{S2}=\underset{F_{S2}\left(t\right)}{\max} \{ [b_{2}\ln\left(\text{e}+\beta_{S}\right)F_{S2}\left(t\right)-\frac{c_{2}}{2}F_{S2}^{2}\left(t\right)+C_{O}+\left.lx_{S2}\left(t\right)\right]+\left.\frac{\partial V_{S2}}{\partial x_{S2}}\left[a_{2}F_{S2}\left(t\right)-\delta x_{S2}\left(t\right)\right]\right\}$$ (16)

Under the sharing information mode, the HJB equation of the social welfare function of the government and corporations are:


$$\rho V_{I1}=\underset{F_{I1}\left(t\right)}{\max} \{ [\left(b_{1}+b_{I}\right)F_{I1}\left(t\right)-\frac{c_{1}}{2}F_{I1}^{2}\left(t\right)-C_{I}-C_{O}+\left.lx_{I1}\left(t\right)\right]+\left.\frac{\partial V_{I1}}{\partial x_{I1}}\left[a_{1}F_{I1}\left(t\right)-\delta x_{I1}\left(t\right)\right]\right\}$$ (17)

$$\rho V_{I2}=\underset{F_{I2}\left(t\right)}{\max} \{ [\left(b_{2}+b_{I}\right)F_{I2}\left(t\right)-\frac{c_{2}}{2}F_{I2}^{2}\left(t\right)-C_{I}+C_{O}+\left.lx_{I2}\left(t\right)\right]+\left.\frac{\partial V_{I2}}{\partial x_{I2}}\left[a_{2}F_{I2}\left(t\right)-\delta x_{I2}\left(t\right)\right]\right\}$$ (18)

### 4.2. Result of equilibrium

Proposition 1: Under the individual action mode, the extent of efforts to control forest pests and diseases, and social benefits of government and corporations are respectively (the specific solving procedure is shown in S1 File):


$$F_{A1}^{*}\left(t\right)=\frac{b_{1}}{c_{1}}+\frac{a_{1}}{c_{1}}\frac{l}{\rho +\delta }$$ (19)

$$F_{A2}^{*}\left(t\right)=\frac{b_{2}}{c_{2}}+\frac{a_{2}}{c_{2}}\frac{l}{\rho +\delta }$$ (20)

$$V_{A1}^{*}=\frac{l}{\rho +\delta }x_{A1}+\frac{1}{\rho }b_{1}\left(\frac{b_{1}}{c_{1}}+\frac{a_{1}}{c_{1}}\frac{l}{\rho +\delta }\right)-\frac{c_{1}}{2}\frac{1}{\rho }{\left(\frac{b_{1}}{c_{1}}+\frac{a_{1}}{c_{1}}\frac{l}{\rho +\delta }\right)}^{2}-\frac{1}{\rho }C_{O}+\frac{1}{\rho }\frac{l}{\rho +\delta }a_{1}\left(\frac{b_{1}}{c_{1}}+\frac{a_{1}}{c_{1}}\frac{l}{\rho +\delta }\right)$$ (21)

$$V_{A2}^{\text{*}}=\frac{l}{\rho +\delta }x_{A2}+\frac{1}{\rho }b_{2}\left(\frac{b_{2}}{c_{2}}+\frac{a_{2}}{c_{2}}\frac{l}{\rho +\delta }\right)-\frac{c_{2}}{2}\frac{1}{\rho }{\left(\frac{b_{2}}{c_{2}}+\frac{a_{2}}{c_{2}}\frac{l}{\rho +\delta }\right)}^{2}+\frac{1}{\rho }C_{O}+\frac{1}{\rho }\frac{l}{\rho +\delta }a_{2}\left(\frac{b_{2}}{c_{2}}+\frac{a_{2}}{c_{2}}\frac{l}{\rho +\delta }\right)$$ (22)

Proposition 2: Under the sharing scarce resources mode, the extent of efforts to control forest pests and diseases, and social benefits of government and corporations are respectively (the specific solving procedure is shown in S2 File):


$$F_{S1}^{*}\left(t\right)=\frac{b_{1}}{c_{1}+c_{S}}+\frac{a_{1}+a_{S}}{c_{1}+c_{S}}\frac{l}{\rho +\delta }$$ (23)

$$F_{S2}^{*}\left(t\right)=\frac{b_{2}\ln\left(\text{e}+\beta_{S}\right)}{c_{2}}+\frac{a_{2}}{c_{2}}\frac{l}{\rho +\delta }$$ (24)

$$\begin{array}{l} V_{S1}^{*}=\frac{l}{\rho +\delta }x_{S1}+\frac{1}{\rho }b_{1}\left(\frac{b_{1}}{c_{1}+c_{S}}+\frac{a_{1}+a_{S}}{c_{1}+c_{S}}\frac{l}{\rho +\delta }\right)-\frac{1}{\rho }\frac{c_{1}+c_{S}}{2}{\left(\frac{b_{1}}{c_{1}+c_{S}}+\frac{a_{1}+a_{S}}{c_{1}+c_{S}}\frac{l}{\rho +\delta }\right)}^{2}-\frac{1}{\rho }C_{O}\\ +\frac{1}{\rho }\frac{l}{\rho +\delta }\left(a_{1}+a_{S}\right)\left(\frac{b_{1}}{c_{1}+c_{S}}+\frac{a_{1}+a_{S}}{c_{1}+c_{S}}\frac{l}{\rho +\delta }\right) \end{array}$$ (25)

$$\begin{array}{l} V_{S2}^{\text{*}}=\frac{l}{\rho +\delta }x_{S2}+\frac{1}{\rho }b_{2}\ln\left(\text{e}+\beta_{S}\right)\left[\frac{b_{2}\ln\left(\text{e}+\beta_{S}\right)}{c_{2}}+\frac{a_{2}}{c_{2}}\frac{l}{\rho +\delta }\right]-\frac{c_{2}}{2}\frac{1}{\rho }{\left[\frac{b_{2}\ln\left(\text{e}+\beta_{S}\right)}{c_{2}}+\frac{a_{2}}{c_{2}}\frac{l}{\rho +\delta }\right]}^{2}\\ +\frac{1}{\rho }C_{O}+\frac{1}{\rho }\frac{l}{\rho +\delta }a_{2}\left[\frac{b_{2}\ln\left(\text{e}+\beta_{S}\right)}{c_{2}}+\frac{a_{2}}{c_{2}}\frac{l}{\rho +\delta }\right] \end{array}$$ (26)

Conclusion 1: The greater the reputation gained from sharing scarce resources, the greater the effort of government in controlling forest pests and diseases. The more significant the role of scarce resources in corporate governance of forest pests and diseases, the greater the effort of corporations in controlling forest pests and diseases.

Proposition 3: Under the sharing information mode, the extent of efforts to control forest pests and diseases, and social benefits of government and corporations are respectively (the specific solving procedure is shown in S3 File):


$$F_{I1}^{*}\left(t\right)=\frac{b_{1}+b_{I}}{c_{1}}+\frac{a_{1}}{c_{1}}\frac{l}{\rho +\delta }$$ (27)

$$F_{I2}^{*}\left(t\right)=\frac{b_{2}+b_{I}}{c_{2}}+\frac{a_{2}}{c_{2}}\frac{l}{\rho +\delta }$$ (28)

$$\begin{array}{l} V_{I1}^{*}=\frac{l}{\rho +\delta }x_{I1}+\frac{1}{\rho }\left(b_{1}+b_{I}\right)\left(\frac{b_{1}+b_{I}}{c_{1}}+\frac{a_{1}}{c_{1}}\frac{l}{\rho +\delta }\right)-\frac{c_{1}}{2}\frac{1}{\rho }{\left(\frac{b_{1}+b_{I}}{c_{1}}+\frac{a_{1}}{c_{1}}\frac{l}{\rho +\delta }\right)}^{2}-\frac{1}{\rho }C_{I}-\frac{1}{\rho }C_{O}\\ +\frac{\partial V_{I1}}{\partial x_{I1}}\frac{1}{\rho }a_{1}\left(\frac{b_{1}+b_{I}}{c_{1}}+\frac{a_{1}}{c_{1}}\frac{l}{\rho +\delta }\right) \end{array}$$ (29)

$$\begin{array}{l} V_{I2}^{\text{*}}=\frac{l}{\rho +\delta }x_{I2}+\frac{1}{\rho }\left(b_{2}+b_{I}\right)\left(\frac{b_{2}+b_{I}}{c_{2}}+\frac{a_{2}}{c_{2}}\frac{l}{\rho +\delta }\right)-\frac{c_{2}}{2}\frac{1}{\rho }{\left(\frac{b_{2}+b_{I}}{c_{2}}+\frac{a_{2}}{c_{2}}\frac{l}{\rho +\delta }\right)}^{2}-\frac{1}{\rho }C_{I}+\frac{1}{\rho }C_{O}\\ +\frac{l}{\rho +\delta }\frac{1}{\rho }a_{2}\left(\frac{b_{2}+b_{I}}{c_{2}}+\frac{a_{2}}{c_{2}}\frac{l}{\rho +\delta }\right) \end{array}$$ (30)

Conclusion 2: The greater the enhancement in the efficiency of forest pest and disease management through information sharing, the greater the effort by both government and corporations in the governance of forest pests and diseases.

### 4.3. Numerical analysis

In order to describe in more detail the changes in social utility of governments and enterprises in the process of forest pests and diseases control, this paper adopts the method of numerical analysis. The following assumptions are made for relevant parameters:

In the process of managing forest pest diseases, “the cost of the government sharing scarce resources with enterprises” is equivalent to “the cost of information sharing between the government and enterprises,” primarily for the following reasons. First, information as a resource: In modern economics and management sciences, information is considered a crucial resource. In the management of forest pest diseases, timely and accurate information about the occurrence, development trends, and effective control measures of pest diseases is extremely valuable. When the government shares this type of information with enterprises, it essentially constitutes a form of resource sharing. Second, the composition of costs: Whether sharing physical resources or information resources, the costs involved typically include collection, processing, transmission, and protection. Information sharing requires special consideration of data accuracy, security, and transmission efficiency, all of which incur specific costs [1]. Third, efficiency and effectiveness: Effective information sharing can greatly enhance the timeliness and accuracy of pest disease response measures, thereby reducing potential additional economic losses due to delays or erroneous decisions. In the long run, the costs invested in information sharing can be compensated by the reduction of resource waste and increased prevention and control costs. Therefore, from an economic and management perspective, the act of the government sharing scarce resources (in this case, critical information) with businesses is essentially the cost of information sharing. This type of sharing is not merely a simple data exchange but involves a complex process that includes building trust, creating and maintaining cooperative mechanisms. Therefore, this paper hypothesizes that cost *c*_*S*_ of government sharing of scarce resources is 3, and costs *C*_*I*_ of information sharing is 3.

In the process of joint forest pest and disease management by governments and enterprises, “the increase in forest pest and disease management benefits from information sharing” is less than “the cost of information sharing,” mainly due to the following two aspects. First, the quality and effectiveness of information. If the shared information is not accurate, timely, or complete, its practical help in managing forest pests and diseases is limited, which may lead to the actual benefits of information sharing failing to meet expectations. The quality of information directly affects the effectiveness of decision-making, and low-quality information can lead to incorrect decisions, increasing the risk of management errors. Second, difficulties in application and implementation. Even with sufficient information sharing, technical, financial, or coordination difficulties encountered by government and enterprises in applying this information for specific pest and disease management can also prevent the actual benefits from reaching expectations [8]. For example, the technology for managing pests and diseases may require specific equipment or knowledge, which might not be readily accessible or implementable in practice. Therefore, this paper hypothesizes that increased benefits *b*_*I*_ of forest pest and disease management from information sharing is 2.5.

“The unit reputation gained by the government or enterprises from managing forest pest diseases exceeds the reputation gained by the government from sharing scarce resources,” mainly for the following reasons. First, the visibility of direct contribution and social impact: Managing forest pest diseases is an action that has a direct and positive impact on the environment, which is tangible, such as improvements in forest conditions and the protection of biodiversity [9]. The public can directly observe or learn about these outcomes through the media, hence, governments or enterprises engaged in management activities tend to gain higher social recognition and reputation. In contrast, although sharing scarce resources is also a positive act, its impact is relatively indirect and may not be easily recognized and evaluated by the public. Second, the perception of urgency and importance: Forest pest diseases are directly related to global challenges such as environmental issues, climate change, and biodiversity, areas that are highly prioritized by the public. Therefore, efforts by governments or enterprises in this area are seen as addressing global, urgent problems, which makes it easier to earn public praise and high evaluation. In comparison, although resource sharing plays a crucial role in improving efficiency and promoting cooperation, its contribution to solving global issues may not be as direct and significant as that of managing forest pest diseases, thus it might yield less reputation gain. Therefore, this paper hypothesizes that the reputation *a*_1_,*a*_2_ gained by government or enterprises for managing forest pests and diseases at a unit level is 2, and the reputation *a*_*S*_ for the government due to sharing of scarce resources is 1.2.

The reasons for the government to grant substantial carbon compensation to enterprises to stimulate their participation in managing forest pest and disease are primarily reflected in the following aspects. First, managing forest pests and diseases requires substantial input, including but not limited to research costs, human resource costs, and physical resource investment. As profit-oriented organizations, enterprises often need to consider costs and benefits when participating in social and environmental projects [10]. Adequate carbon compensation can help cover these costs, thus motivating them to allocate resources for management. Second, the nature of carbon compensation is an economic incentive mechanism that provides financial rewards for actions that reduce greenhouse gas emissions or increase carbon sequestration. In forest pest and disease management, effective control not only protects the forest ecosystem but also enhances the forest’s carbon sequestration function, that is, absorbing more carbon dioxide. Therefore, providing carbon compensation to incentivize enterprises to actively participate in forest conservation is an effective means of using market mechanisms to promote environmental governance. Third, enterprises weigh the risks and benefits when deciding whether to participate in managing forest pests and diseases. The management activities themselves may involve high risks and uncertainties, such as the uncertainty of pest control effectiveness and the instability of long-term investments. Reasonable carbon compensation can provide enterprises with clear profit expectations, helping them balance risks and benefits, and encouraging them to take action. Therefore, this paper hypothesizes that government carbon offsets *C*_*o*_ to enterprises is 10.

At the same time, the following assumptions are made about insignificant parameters in this paper. The discount rate *ρ* that occurs over time is 0.9. Decay *δ* of reputation is 0.1. Extent *β*_S_ to which scarce resources help firms manage forest pests and diseases is 1.5. The positive effects *l* of reputation is 1. Also, if it is in the unit state, i.e., *x* =1.

When the cost *c*_1_, *c*_2_ to government or social force of controlling forest pests and diseases at a unit level is 2, this article can calculate the social benefits of government:


$$V_{A1}^{*}=-10.111+0.278{\left(b_{1}+2\right)}^{2}$$ (31)

$$V_{S1}^{*}=-10.111+0.111{\left(b_{1}+3.2\right)}^{2}$$ (32)

$$V_{I1}^{*}=-13.444+0.278{\left(b_{1}+4.5\right)}^{2}$$ (33)

**Fig 3:**
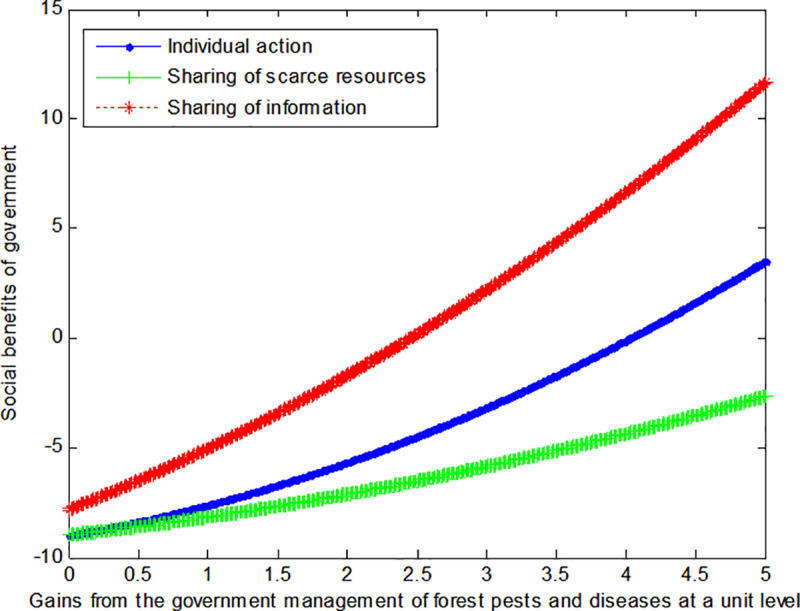
Impact of gains of government on social welfare.

The following graph (named Fig 3) can also be produced:

When the cost *c*_1_, *c*_2_ to government or social force of controlling forest pests and diseases at a unit level is 4, this article can calculate the social benefits of government:


$$V_{A1}^{*}=-10.111+0.139{\left(b_{1}+2\right)}^{2}$$ (34)

$$V_{S1}^{*}=-10.111+0.079{\left(b_{1}+3.2\right)}^{2}$$ (35)

$$V_{I1}^{*}=-13.444+0.139{\left(b_{1}+4.5\right)}^{2}$$ (36)

**Fig 4:**
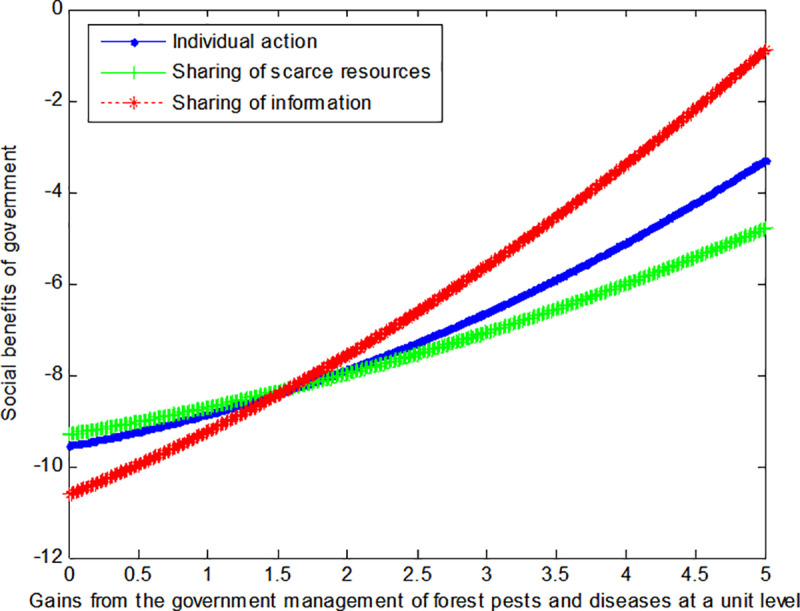
Impact of gains of government on social welfare.

The following graph (named Fig 4) can also be produced:

Conclusion 3: If the cost of controlling forest pests and diseases by government units is high, and the benefits from such control are low, then the sharing of scarce resources can enable the government to achieve maximum benefits. Conversely, the sharing of information can enable the government to achieve maximum benefits if the situation is reversed.

When the cost *c*_1_, *c*_2_ to government or social force of controlling forest pests and diseases at a unit level is 2, this article can calculate the social benefits of enterprises:


$$V_{A2}^{\text{*}}=12.111+0.278{\left(b_{2}+2\right)}^{2}$$ (37)

$$V_{S2}^{\text{*}}=12.111+0.278{\left(1.44b_{2}+2\right)}^{2}$$ (38)

$$V_{I2}^{\text{*}}=8.778+0.278{\left(b_{2}+4.5\right)}^{2}$$ (39)

**Fig 5:**
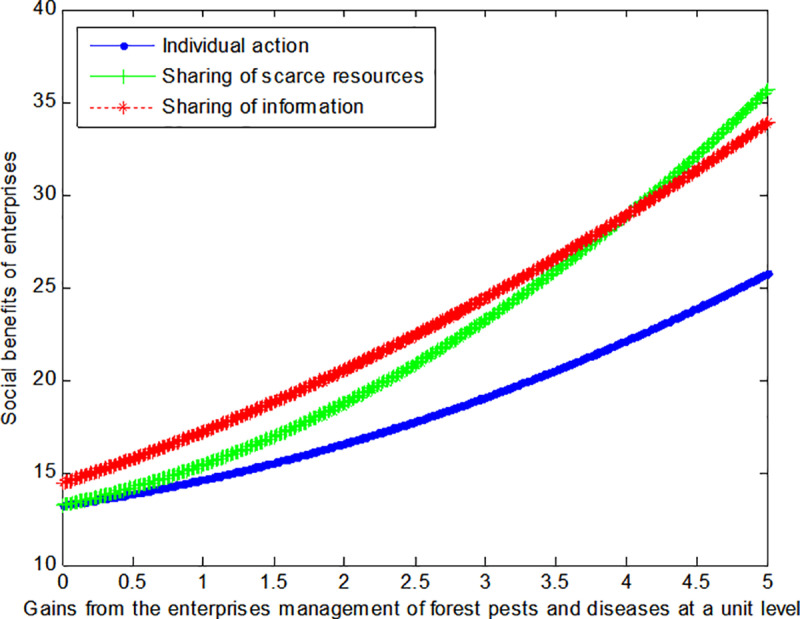
Impact of gains of enterprises on social welfare.

The following graph (named Fig 5) can also be produced:

When the cost *c*_1_, *c*_2_ to government or social force of controlling forest pests and diseases at a unit level is 4, this article can calculate the social benefits of enterprises:


$$V_{A2}^{\text{*}}=12.111+0.139{\left(b_{2}+2\right)}^{2}$$ (40)

$$V_{S2}^{\text{*}}=12.111+0.139{\left(1.44b_{2}+2\right)}^{2}$$ (41)

$$V_{I2}^{\text{*}}=8.778+0.139{\left(b_{2}+4.5\right)}^{2}$$ (42)

**Fig 6:**
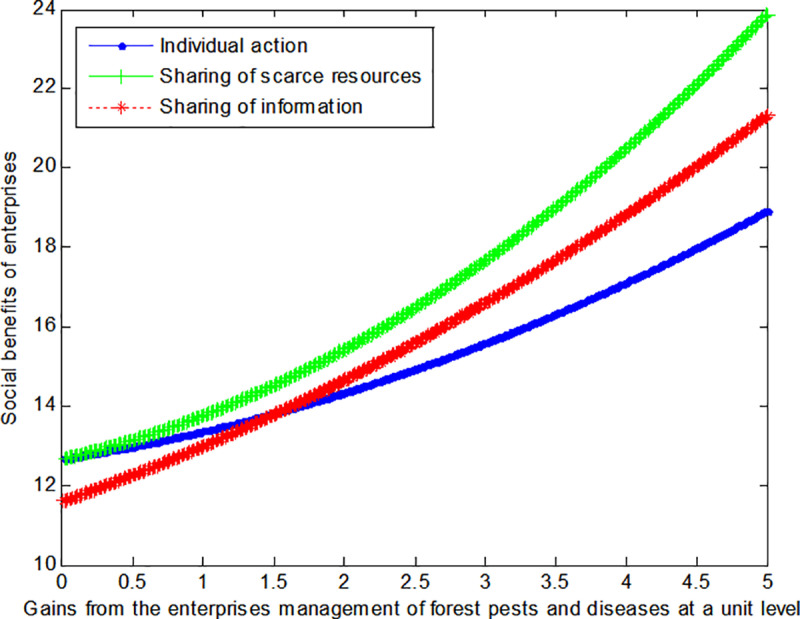
Impact of gains of enterprises on social welfare.

The following graph (named Fig 6) can also be produced:

Conclusion 4: If the cost incurred and the benefits derived from corporate-level control of forest pests and diseases are modest, then an information sharing mode can enable corporations to achieve maximum benefits. Conversely, the scarce resource sharing mode can enable corporations to realize maximum benefits when the opposite is true.

## 5. Discussion

According to Conclusion 1, the greater the role of scarce resources in corporate governance of forest pest and disease management, the more intensive the efforts in this governance. This echoes the findings of Fang et al. [2], who suggested that reducing the flight path and the number of turns in helicopter pesticide application could lower operational costs and thus be advantageous in managing forest pests and diseases. Scarce resources shared by the government might include financial aid, expertise, technological support, workforce training, and more, which substantially affect a company’s capability and willingness to address forest pest and disease issues. Here are several ways this type of resource sharing could influence corporate governance efforts. First, when governments provide funding or other forms of support, companies may be more motivated to engage in pest and disease management, as these resources can help to cover the costs incurred, thereby alleviating the financial burden on the companies. Second, the provision of scarce resources can aid companies to acquire the necessary technology and materials to combat forest pests and diseases effectively, such as cutting-edge monitoring and early warning systems, biological control techniques, or more efficient chemical treatment methods. Third, the sharing of expertise and effective workforce training can heighten the professional capacity of companies to tackle forest pests and diseases, making management more scientific and efficient. Fourth, government support can incentivize companies to invest in researching and developing new management approaches, including eco-friendly solutions like biological control and the cultivation of pest-resistant tree species. Should the government provide scarce resources that are beneficial to companies, it is likely that firms will increase their efforts in managing forest pests and diseases as they utilize these resources. However, it is also necessary to pay attention to the efficient management and allocation of resources, to ensure that they are not misused and that their positive impact on forest pest and disease management is maximized.

The following management implications can be drawn from Conclusion 1. If the government can gain a higher reputation by sharing scarce resources, then it will invest more effort in controlling forest pests and diseases. Therefore, the government should actively establish and optimize resource sharing mechanisms, such as inter-departmental collaboration platforms, expert team sharing, and advanced equipment and technology sharing, to improve governance efficiency and effectiveness. At the same time, the government should strengthen publicity by showcasing successful cases of its governance outcomes, enhancing public and stakeholder trust and support, thereby further motivating the government’s enthusiasm and sense of responsibility. In forest pest and disease governance, if scarce resources play a more significant role in the process, then companies will invest greater effort in controlling pests and diseases [19]. Therefore, companies should actively seek cooperation with the government, other companies, and research institutions to establish resource sharing mechanisms and share advanced technologies and equipment. This not only helps to reduce governance costs and improve governance effectiveness but also enhances the company’s social reputation and sense of responsibility. Through these measures, companies can achieve a win-win situation in terms of economic benefits and environmental protection.

According to Conclusion 2, the greater the benefit improvement in managing forest pests and diseases through information sharing, the more intensive the efforts by both governments and corporations in managing forest pests and diseases. This stands in contrast to the study by León-Bañuelos et al. [4], which posited that the presence of harmful organisms and parasitic plants in forests could be analyzed through the use of unmanned aerial vehicles and algorithms. León-Bañuelos et al. [4] focused on enhancing forest pest and disease management through technological means, whereas the present study concentrates on management approaches. The rationale behind Conclusion 2 is as follows. Information sharing contributes to a more comprehensive understanding among all stakeholders regarding the occurrence, development, and consequences of pest and disease outbreaks. This heightened awareness prompts governments and corporations to acknowledge the severity and urgency of forest pests and diseases, leading to more proactive measures. Through information sharing, different stakeholders can more effectively coordinate their activities and resources for joint action. Collaboration facilitates more unified and focused governance efforts, thereby improving overall efficiency. Information sharing enables governments and corporations to monitor the trends of forest pests and diseases in real-time, allowing for swift action to prevent their spread or exacerbation early on. Improved information flow aids decision-makers in making data- and science-based decisions, reducing the costs of trial and error and further motivating the input of more resources into forest pest and disease management by both governments and corporations. When information is freely shared, trust between governments, corporations, and the public is enhanced. This transparency fosters more positive policy support and higher public engagement. The shared information offers opportunities for learning and innovation. Understanding the complex dynamics of forest pests and diseases can inspire new management methods, technologies, and more effective strategies. Information sharing is pivotal for accurately identifying resource needs, allowing scarce resources to be allocated where they are most needed and optimizing overall resource distribution. Hence, information sharing is crucial for enhancing the efficiency of forest pest and disease management. When governments and corporations see that sharing information significantly enhances management efficacy, they are more inclined to increase their input, including time, funds, and technology, to minimize the adverse impacts of pests and diseases.

The following management implications can be drawn from Conclusion 2. Information sharing plays a significant role in enhancing the efficiency of forest pest and disease management. Therefore, the government and enterprises should increase the intensity of information sharing in forest pest and disease control, establishing and improving information sharing platforms to ensure that all levels of departments and stakeholders can timely access and exchange monitoring data, prevention and control measures, and the latest research findings [20]. Through efficient information flow, the scientific nature of decision-making and response speed can be improved, effectively enhancing overall governance efficiency. Furthermore, the government and enterprises should work together to formulate and promote standards and norms for information sharing, improving the quality and usability of information. The government can introduce relevant policies to encourage and support information sharing cooperation among enterprises, providing necessary technical support and training, while enterprises should actively participate in information sharing, enhancing their data management capabilities and technical skills, jointly building an efficient forest pest and disease management ecosystem.

According to Conclusion 3, when the cost of the government’s per-unit governance of forest pests and diseases is high, and the benefits of per-unit governance are low, the scarce resource sharing mode can yield maximum benefits for the government. This bears similarities to, but is not identical with, the study by Garnas et al. [12], which posited that cost-sharing, the growth and maintenance of resources and capabilities, and a more comprehensive research plan are crucial for the long-term success of biological control. This study, however, elucidates under which conditions the sharing of scarce resources is necessary through an analysis. The reasons behind Conclusion 3 are as follows. First, by sharing resources with businesses or other governmental entities, the government can distribute the high costs required to individually address forest pests and diseases. This approach allows for the allocation of reasonable costs while achieving governance over a wider area. Second, the resource-sharing mode facilitates economies of scale, resulting in a decrease in the cost per unit of governance as the scale increases. Governments can reduce unit costs through mass purchasing and deployment. Third, resource sharing encourages collaboration and cooperation between different entities. Efficient cooperation not only saves costs economically but also enhances the overall efficiency and success rate of forest pest and disease management through the combination of collective wisdom and experience. Fourth, sharing research and development outcomes and other technologies can help governments identify and address forest pest and disease issues more precisely, reducing unnecessary resource wastage and improving governance outcomes. Fifth, resource sharing enables governments to concentrate resources in the areas and times where they are most needed, rather than over-investing at inappropriate times or locations, thereby enhancing the governance benefits per unit of resource. If scarce resources are managed effectively, for example, by properly setting governance priorities, ensuring concentrated application of resources in key areas, and implementing scientific management methods, this resource-sharing mode can allow the government to attain relatively greater net benefits in forest pest and disease management, even under unfavorable conditions of high costs and low benefits.

The following management implications can be drawn from Conclusion 3. If the cost of controlling forest pests and diseases by government agencies is high while the benefits are low, then maximizing governance effectiveness through the sharing of scarce resources can be achieved. The government should establish cross-departmental and cross-regional cooperation mechanisms, reasonably allocate and share limited financial, technical, and human resources, reduce redundant investments, and enhance resource utilization efficiency, thereby achieving better governance outcomes under constrained conditions [21]. Conversely, if the cost of controlling forest pests and diseases by government agencies is low while the benefits are high, then further improving governance effectiveness through information sharing can be realized. The government should actively develop and improve information sharing platforms to ensure that all relevant departments and stakeholders can promptly access accurate pest and disease information, enhancing the scientific nature of decision-making and response speed, thereby optimizing resource allocation and improving governance efficiency.

According to Conclusion 4, if the cost associated with corporate governance of forest pests and diseases and the benefits derived from it are small, an information sharing mode can yield the maximum benefit for businesses. This has similarities to, but is not identical with, the study by Qin [7], which suggested that establishing a platform for analyzing the correlation between meteorological conditions and the dynamics of forest harmful organisms could improve the forecasting and early warning of pests. However, this paper focuses on the conditions under which information sharing is necessary. The reasons behind Conclusion 4 are as follows. First, information sharing can reduce redundant research and labor among different businesses, such as in the monitoring and detection of pest and disease occurrences and patterns. This sharing reduces the repetitiveness of work and lowers overall costs. Second, sharing information helps to quickly identify and adopt the best governance methods. Companies do not have to invest resources in experimentation alone but can leverage the successful cases and practical experiences shared by other businesses or government. Third, through information sharing, businesses can gain a more accurate understanding of the distribution and development trends of forest pests and diseases, making response measures more precise and effective while avoiding resource wastage. Fourth, information sharing among companies can lead to the formation of collaborative alliances to jointly address pest and disease issues. This united front not only can enhance governance efficacy but may also produce synergistic advantages, such as reducing costs through bulk purchasing of goods and services collectively [18]. Fifth, information sharing enables businesses to be informed of risks and potential problems in a timely manner, allowing for immediate measures to reduce or prevent damage. Such early intervention can mitigate potential long-term costs.

The following management implications can be drawn from Conclusion 4. If the costs incurred and benefits obtained by enterprises in controlling forest pests and diseases are relatively limited, then maximizing governance effectiveness through an information sharing model can be achieved. Enterprises should establish and utilize efficient information sharing platforms to share pest and disease monitoring data, control technologies, and best practices with peers and other relevant parties, reducing information asymmetry, enhancing the scientific nature of decision-making and control effectiveness, thereby achieving optimal governance goals with limited resources. Conversely, if enterprises face high costs and low benefits in controlling forest pests and diseases, then maximizing governance effectiveness through the sharing of scarce resources can be realized. Enterprises should strengthen cooperation with other enterprises, research institutions, and the government, sharing technical equipment, specialized talents, and financial resources, reducing redundant investments, and enhancing resource utilization efficiency, thereby achieving better control outcomes and economic benefits under high-cost conditions.

Of course, this study has certain limitations. Firstly, the limitations of model assumptions. Any mathematical model, including the differential game model, needs to be constructed based on certain assumptions. These assumptions may simplify the complex environmental, economic, and social factors in the real world, leading to discrepancies between the model results and actual conditions. For example, assuming that all participants can make decisions rationally and completely, or that all relevant information is transparent and can be obtained instantly, may not fully align with reality. Secondly, the difficulty in data acquisition. To accurately construct and run such a model, a large amount of empirical data is needed to support parameter settings and model validation. However, obtaining comprehensive, timely, and accurate data in areas such as forest pest and disease management and carbon trading is quite challenging, often due to limitations in geography, time, or resources. Thirdly, the neglect of individual differences. In practical applications, different individuals or organizations have varying interests, capabilities, and motivations in forest management and carbon trading. The differential game model may fail to adequately capture these differences between individuals, which are crucial for formulating effective management strategies.

## 6. Conclusion

Pests and diseases cause significant damage to forest ecosystems, necessitating effective protection by both governments and corporations. In a carbon compensation context, the government needs to award certain carbon emission rights to businesses involved in forest pest and disease management. Considering the modes of collaboration between governments and businesses in managing forest pests and diseases, namely, individual action, scarce resource sharing, and information sharing, this paper constructs differential game models for these three modes, derives equilibrium outcomes, and conducts comparative analyses. The study concludes that if the cost of government per-unit governance of forest pests and diseases is high and the benefits of per-unit governance are low, then the scarce resource sharing mode can yield maximum benefits for the government. Conversely, the information sharing mode can provide the greatest benefits for the government. If the cost and benefits of corporate per-unit governance of forest pests and diseases are low, then the information sharing mode can yield the maximum benefits for corporations; conversely, the scarce resource sharing mode can provide the greatest benefits for corporations.

Forest pest management and carbon trading are both hot topics in the fields of environmental science and ecological management. Numerous studies have explored how carbon trading mechanisms can promote forest health and sustainable management. If the differential game approach adopted in this paper has been widely applied in existing literature, then these methods themselves do not bring new breakthroughs. Therefore, the theme of this paper does not possess significant innovativeness. In future research, this study will analyze forest governance from a completely new perspective or improve existing research methods to enhance related innovativeness.

The research presented in this paper can be extended in future work. For instance, it assumes that scarce resource sharing incurs additional costs for the government but also earns it extra reputation; such sharing increases the benefits for corporations; and information sharing improves governance efficiency for both the government and corporations. These assumptions could be removed for further exploration in future research. Additionally, some gaps identified in this study may be addressed in subsequent investigations. First, specific criteria for adopting different forest pest and disease management modes by the government and corporations under varying conditions should be established. Second, the findings of forest pest and disease management under carbon compensation could be translated into practical policy recommendations applicable in regions severely affected by forest pests and diseases. Third, during the pest and disease management process in various regions, the government and corporations should determine the sequence of their respective research actions instead of proceeding simultaneously.

## Supporting information

S1 File Proof of (19) - (22). (DOCX)

S2 File Proof of (23) - (26). (DOCX)

S3 File Proof of (27) - (30). (DOCX)
