# Supplementary material for: Individual action, sharing scarce resources, sharing information? A study on how to effectively manage forest pests and diseases based on carbon trading
Source: PLoS One. 2025 Apr 28;20(4):e0322237. doi: 10.1371/journal.pone.0322237 (PMC12036916; doi:10.1371/journal.pone.0322237)
Supplement: S1 File — (DOCX) [file pone.0322237.s001.docx]

**Supporting information 1**

Take the derivatives of *F_A_*_1_ with respect to (13), and take the derivatives of *F_A_*_2_ with respect to (14), and set them equal to zero, we can get:

$F_{A1}^{*}\left(t\right)=\frac{b_{1}}{c_{1}}+\frac{a_{1}}{c_{1}}\frac{\partial V_{A1}}{\partial x_{A1}}$ (43)

$F_{A2}^{*}\left(t\right)=\frac{b_{2}}{c_{2}}+\frac{a_{2}}{c_{2}}\frac{\partial V_{A2}}{\partial x_{A2}}$ (44)

Substituting (43) into (13) and substituting (44) into (14), we can get:

$\rho V_{A1}=b_{1}\left(\frac{b_{1}}{c_{1}}+\frac{a_{1}}{c_{1}}\frac{\partial V_{A1}}{\partial x_{A1}}\right)-\frac{c_{1}}{2}\left(\frac{b_{1}}{c_{1}}+\frac{a_{1}}{c_{1}}\frac{\partial V_{A1}}{\partial x_{A1}}\right)^{2}-C_{O}+lx_{A1}\left(t\right)+\frac{\partial V_{A1}}{\partial x_{A1}}\left[a_{1}\left(\frac{b_{1}}{c_{1}}+\frac{a_{1}}{c_{1}}\frac{\partial V_{A1}}{\partial x_{A1}}\right)-\delta x_{A1}\left(t\right)\right]$ (45)

$\rho V_{A2}=b_{2}\left(\frac{b_{2}}{c_{2}}+\frac{a_{2}}{c_{2}}\frac{\partial V_{A2}}{\partial x_{A2}}\right)-\frac{c_{2}}{2}\left(\frac{b_{2}}{c_{2}}+\frac{a_{2}}{c_{2}}\frac{\partial V_{A2}}{\partial x_{A2}}\right)^{2}+C_{O}+lx_{A2}\left(t\right)+\frac{\partial V_{A2}}{\partial x_{A2}}\left[a_{2}\left(\frac{b_{2}}{c_{2}}+\frac{a_{2}}{c_{2}}\frac{\partial V_{A2}}{\partial x_{A2}}\right)-\delta x_{A2}\left(t\right)\right]$(46)

Let $V_{A1}^{*}=k_{1}x_{A1}+k_{2}$, $V_{A2}^{\text{*}}=k_{3}x_{A2}+k_{4}$, wherein, *k*_1_, *k*_2_, *k*_3_ and *k*_4_ are all constants. The parameters of the optimal social welfare function can be obtained by calculation as follows:

$\left\{\begin{matrix} k_{1}=\frac{l}{\rho+\delta} \\ k_{2}=\frac{1}{\rho}b_{1}\left(\frac{b_{1}}{c_{1}}+\frac{a_{1}}{c_{1}}\frac{l}{\rho+\delta}\right)-\frac{c_{1}}{2}\frac{1}{\rho}\left(\frac{b_{1}}{c_{1}}+\frac{a_{1}}{c_{1}}\frac{l}{\rho+\delta}\right)^{2}-\frac{1}{\rho}C_{O}+\frac{1}{\rho}\frac{l}{\rho+\delta}a_{1}\left(\frac{b_{1}}{c_{1}}+\frac{a_{1}}{c_{1}}\frac{l}{\rho+\delta}\right) \end{matrix}\right.$ (47)

$\left\{\begin{matrix} k_{3}=\frac{l}{\rho+\delta} \\ k_{4}=\frac{1}{\rho}b_{2}\left(\frac{b_{2}}{c_{2}}+\frac{a_{2}}{c_{2}}\frac{l}{\rho+\delta}\right)-\frac{c_{2}}{2}\frac{1}{\rho}\left(\frac{b_{2}}{c_{2}}+\frac{a_{2}}{c_{2}}\frac{l}{\rho+\delta}\right)^{2}+\frac{1}{\rho}C_{O}+\frac{1}{\rho}\frac{l}{\rho+\delta}a_{2}\left(\frac{b_{2}}{c_{2}}+\frac{a_{2}}{c_{2}}\frac{l}{\rho+\delta}\right) \end{matrix}\right.$ (48)

Therefore, it can be concluded that:

$V_{A1}^{*}=\frac{l}{\rho+\delta}x_{A1}+\frac{1}{\rho}b_{1}\left(\frac{b_{1}}{c_{1}}+\frac{a_{1}}{c_{1}}\frac{l}{\rho+\delta}\right)-\frac{c_{1}}{2}\frac{1}{\rho}\left(\frac{b_{1}}{c_{1}}+\frac{a_{1}}{c_{1}}\frac{l}{\rho+\delta}\right)^{2}-\frac{1}{\rho}C_{O}+\frac{1}{\rho}\frac{l}{\rho+\delta}a_{1}\left(\frac{b_{1}}{c_{1}}+\frac{a_{1}}{c_{1}}\frac{l}{\rho+\delta}\right)$ (49)

$V_{A2}^{\text{*}}=\frac{l}{\rho+\delta}x_{A2}+\frac{1}{\rho}b_{2}\left(\frac{b_{2}}{c_{2}}+\frac{a_{2}}{c_{2}}\frac{l}{\rho+\delta}\right)-\frac{c_{2}}{2}\frac{1}{\rho}\left(\frac{b_{2}}{c_{2}}+\frac{a_{2}}{c_{2}}\frac{l}{\rho+\delta}\right)^{2}+\frac{1}{\rho}C_{O}+\frac{1}{\rho}\frac{l}{\rho+\delta}a_{2}\left(\frac{b_{2}}{c_{2}}+\frac{a_{2}}{c_{2}}\frac{l}{\rho+\delta}\right)$(50)

In this case,

$F_{A1}^{*}\left(t\right)=\frac{b_{1}}{c_{1}}+\frac{a_{1}}{c_{1}}\frac{l}{\rho+\delta}$ (51)

$F_{A2}^{*}\left(t\right)=\frac{b_{2}}{c_{2}}+\frac{a_{2}}{c_{2}}\frac{l}{\rho+\delta}$ (52)
