# Supplementary material for: Individual action, sharing scarce resources, sharing information? A study on how to effectively manage forest pests and diseases based on carbon trading
Source: PLoS One. 2025 Apr 28;20(4):e0322237. doi: 10.1371/journal.pone.0322237 (PMC12036916; doi:10.1371/journal.pone.0322237)
Supplement: S2 File — (DOCX) [file pone.0322237.s002.docx]

**Supporting information 2**

Take the derivatives of *F_S_*_1_ with respect to (15), and take the derivatives of *F_S_*_2_ with respect to (16), and set them equal to zero, we can get:

$F_{S1}^{*}\left(t\right)=\frac{b_{1}}{c_{1}+c_{S}}+\frac{a_{1}+a_{S}}{c_{1}+c_{S}}\frac{\partial V_{S1}}{\partial x_{S1}}$ (53)

$F_{S2}^{*}\left(t\right)=\frac{b_{2}\ln\left(\text{e}+\beta_{S}\right)}{c_{2}}+\frac{a_{2}}{c_{2}}\frac{\partial V_{S2}}{\partial x_{S2}}$ (54)

Substituting (53) into (15) and substituting (54) into (16), we can get:

$\begin{matrix} \rho V_{S1}=b_{1}\left(\frac{b_{1}}{c_{1}+c_{S}}+\frac{a_{1}+a_{S}}{c_{1}+c_{S}}\frac{\partial V_{S1}}{\partial x_{S1}}\right)-\frac{c_{1}+c_{S}}{2}\left(\frac{b_{1}}{c_{1}+c_{S}}+\frac{a_{1}+a_{S}}{c_{1}+c_{S}}\frac{\partial V_{S1}}{\partial x_{S1}}\right)^{2}-C_{O}+lx_{S1}\left(t\right) \\ +\frac{\partial V_{S1}}{\partial x_{S1}}\left[\left(a_{1}+a_{S}\right)\left(\frac{b_{1}}{c_{1}+c_{S}}+\frac{a_{1}+a_{S}}{c_{1}+c_{S}}\frac{\partial V_{S1}}{\partial x_{S1}}\right)-\delta x_{S1}\left(t\right)\right] \end{matrix}$ (55)

$\begin{matrix} \rho V_{S2}=b_{2}\ln\left(\text{e}+\beta_{S}\right)\left[\frac{b_{2}\ln\left(\text{e}+\beta_{S}\right)}{c_{2}}+\frac{a_{2}}{c_{2}}\frac{\partial V_{S2}}{\partial x_{S2}}\right]-\frac{c_{2}}{2}\left[\frac{b_{2}\ln\left(\text{e}+\beta_{S}\right)}{c_{2}}+\frac{a_{2}}{c_{2}}\frac{\partial V_{S2}}{\partial x_{S2}}\right]^{2}+C_{O} \\ +lx_{S2}\left(t\right)+\frac{\partial V_{S2}}{\partial x_{S2}}a_{2}\left[\frac{b_{2}\ln\left(\text{e}+\beta_{S}\right)}{c_{2}}+\frac{a_{2}}{c_{2}}\frac{\partial V_{S2}}{\partial x_{S2}}\right]-\frac{\partial V_{S2}}{\partial x_{S2}}\delta x_{S2}\left(t\right) \end{matrix}$ (56)

Let $V_{S1}^{*}=k_{5}x_{S1}+k_{6}$,$V_{S2}^{\text{*}}=k_{7}x_{S2}+k_{8}$, wherein, *k*_5_, *k*_6_, *k*_7_ and *k*_8_ are all constants. The parameters of the optimal social welfare function can be obtained by calculation as follows:

$\left\{\begin{matrix} k_{5}=\frac{l}{\rho+\delta} \\ k_{6}=\frac{1}{\rho}b_{1}\left(\frac{b_{1}}{c_{1}+c_{S}}+\frac{a_{1}+a_{S}}{c_{1}+c_{S}}\frac{l}{\rho+\delta}\right)-\frac{1}{\rho}\frac{c_{1}+c_{S}}{2}\left(\frac{b_{1}}{c_{1}+c_{S}}+\frac{a_{1}+a_{S}}{c_{1}+c_{S}}\frac{l}{\rho+\delta}\right)^{2}-\frac{1}{\rho}C_{O} \\ +\frac{1}{\rho}\frac{l}{\rho+\delta}\left(a_{1}+a_{S}\right)\left(\frac{b_{1}}{c_{1}+c_{S}}+\frac{a_{1}+a_{S}}{c_{1}+c_{S}}\frac{l}{\rho+\delta}\right) \end{matrix}\right.$ (57)

$\left\{\begin{matrix} k_{7}=\frac{l}{\rho+\delta} \\ k_{8}=\frac{1}{\rho}b_{2}\ln\left(\text{e}+\beta_{S}\right)\left[\frac{b_{2}\ln\left(\text{e}+\beta_{S}\right)}{c_{2}}+\frac{a_{2}}{c_{2}}\frac{l}{\rho+\delta}\right]-\frac{c_{2}}{2}\frac{1}{\rho}\left[\frac{b_{2}\ln\left(\text{e}+\beta_{S}\right)}{c_{2}}+\frac{a_{2}}{c_{2}}\frac{l}{\rho+\delta}\right]^{2}+\frac{1}{\rho}C_{O} \\ +\frac{1}{\rho}\frac{l}{\rho+\delta}a_{2}\left[\frac{b_{2}\ln\left(\text{e}+\beta_{S}\right)}{c_{2}}+\frac{a_{2}}{c_{2}}\frac{l}{\rho+\delta}\right] \end{matrix}\right.$ (58)

Therefore, it can be concluded that:

$\begin{matrix} V_{S1}^{*}=\frac{l}{\rho+\delta}x_{S1}+\frac{1}{\rho}b_{1}\left(\frac{b_{1}}{c_{1}+c_{S}}+\frac{a_{1}+a_{S}}{c_{1}+c_{S}}\frac{l}{\rho+\delta}\right)-\frac{1}{\rho}\frac{c_{1}+c_{S}}{2}\left(\frac{b_{1}}{c_{1}+c_{S}}+\frac{a_{1}+a_{S}}{c_{1}+c_{S}}\frac{l}{\rho+\delta}\right)^{2}-\frac{1}{\rho}C_{O} \\ +\frac{1}{\rho}\frac{l}{\rho+\delta}\left(a_{1}+a_{S}\right)\left(\frac{b_{1}}{c_{1}+c_{S}}+\frac{a_{1}+a_{S}}{c_{1}+c_{S}}\frac{l}{\rho+\delta}\right) \end{matrix}$ (59)

$\begin{matrix} V_{S2}^{\text{*}}=\frac{l}{\rho+\delta}x_{S2}+\frac{1}{\rho}b_{2}\ln\left(\text{e}+\beta_{S}\right)\left[\frac{b_{2}\ln\left(\text{e}+\beta_{S}\right)}{c_{2}}+\frac{a_{2}}{c_{2}}\frac{l}{\rho+\delta}\right]-\frac{c_{2}}{2}\frac{1}{\rho}\left[\frac{b_{2}\ln\left(\text{e}+\beta_{S}\right)}{c_{2}}+\frac{a_{2}}{c_{2}}\frac{l}{\rho+\delta}\right]^{2} \\ +\frac{1}{\rho}C_{O}+\frac{1}{\rho}\frac{l}{\rho+\delta}a_{2}\left[\frac{b_{2}\ln\left(\text{e}+\beta_{S}\right)}{c_{2}}+\frac{a_{2}}{c_{2}}\frac{l}{\rho+\delta}\right] \end{matrix}$(60)

In this case,

$F_{S1}^{*}\left(t\right)=\frac{b_{1}}{c_{1}+c_{S}}+\frac{a_{1}+a_{S}}{c_{1}+c_{S}}\frac{l}{\rho+\delta}$ (61)

$F_{S2}^{*}\left(t\right)=\frac{b_{2}\ln\left(\text{e}+\beta_{S}\right)}{c_{2}}+\frac{a_{2}}{c_{2}}\frac{l}{\rho+\delta}$ (62)
