# Supplementary material for: Individual action, sharing scarce resources, sharing information? A study on how to effectively manage forest pests and diseases based on carbon trading
Source: PLoS One. 2025 Apr 28;20(4):e0322237. doi: 10.1371/journal.pone.0322237 (PMC12036916; doi:10.1371/journal.pone.0322237)
Supplement: S3 File — (DOCX) [file pone.0322237.s003.docx]

**Supporting information 3**

Take the derivatives of *F_I_*_1_ with respect to (17), and take the derivatives of *F_I_*_2_ with respect to (18), and set them equal to zero, we can get:

$F_{I1}^{*}\left(t\right)=\frac{b_{1}+b_{I}}{c_{1}}+\frac{a_{1}}{c_{1}}\frac{\partial V_{I1}}{\partial x_{I1}}$ (63)

$F_{I2}^{*}\left(t\right)=\frac{b_{2}+b_{I}}{c_{2}}+\frac{a_{2}}{c_{2}}\frac{\partial V_{I2}}{\partial x_{I2}}$ (64)

Substituting (63) into (17) and substituting (64) into (18), we can get:

$\begin{matrix} \rho V_{I1}=\left(b_{1}+b_{I}\right)\left(\frac{b_{1}+b_{I}}{c_{1}}+\frac{a_{1}}{c_{1}}\frac{\partial V_{I1}}{\partial x_{I1}}\right)-\frac{c_{1}}{2}\left(\frac{b_{1}+b_{I}}{c_{1}}+\frac{a_{1}}{c_{1}}\frac{\partial V_{I1}}{\partial x_{I1}}\right)^{2}-C_{I}-C_{O}+lx_{I1}\left(t\right) \\ +\frac{\partial V_{I1}}{\partial x_{I1}}\left[a_{1}\left(\frac{b_{1}+b_{I}}{c_{1}}+\frac{a_{1}}{c_{1}}\frac{\partial V_{I1}}{\partial x_{I1}}\right)-\delta x_{I1}\left(t\right)\right] \end{matrix}$ (65)

$\begin{matrix} \rho V_{I2}=\left(b_{2}+b_{I}\right)\left(\frac{b_{2}+b_{I}}{c_{2}}+\frac{a_{2}}{c_{2}}\frac{\partial V_{I2}}{\partial x_{I2}}\right)-\frac{c_{2}}{2}\left(\frac{b_{2}+b_{I}}{c_{2}}+\frac{a_{2}}{c_{2}}\frac{\partial V_{I2}}{\partial x_{I2}}\right)^{2}-C_{I}+C_{O}+lx_{I2}\left(t\right) \\ +\frac{\partial V_{I2}}{\partial x_{I2}}\left[a_{2}\left(\frac{b_{2}+b_{I}}{c_{2}}+\frac{a_{2}}{c_{2}}\frac{\partial V_{I2}}{\partial x_{I2}}\right)-\delta x_{I2}\left(t\right)\right] \end{matrix}$ (66)

Let $V_{I1}^{*}=k_{9}x_{I1}+k_{10}$,$V_{I2}^{\text{*}}=k_{11}x_{I2}+k_{12}$, wherein, *k*_9_, *k*_10_, *k*_11_ and *k*_12_ are all constants. The parameters of the optimal social welfare function can be obtained by calculation as follows:

$\left\{\begin{matrix} k_{9}=\frac{l}{\rho+\delta} \\ k_{10}=\frac{1}{\rho}\left(b_{1}+b_{I}\right)\left(\frac{b_{1}+b_{I}}{c_{1}}+\frac{a_{1}}{c_{1}}\frac{l}{\rho+\delta}\right)-\frac{c_{1}}{2}\frac{1}{\rho}\left(\frac{b_{1}+b_{I}}{c_{1}}+\frac{a_{1}}{c_{1}}\frac{l}{\rho+\delta}\right)^{2}-\frac{1}{\rho}C_{I}-\frac{1}{\rho}C_{O} \\ +\frac{\partial V_{I1}}{\partial x_{I1}}\frac{1}{\rho}a_{1}\left(\frac{b_{1}+b_{I}}{c_{1}}+\frac{a_{1}}{c_{1}}\frac{l}{\rho+\delta}\right) \end{matrix}\right.$ (67)

$\left\{\begin{matrix} k_{11}=\frac{l}{\rho+\delta} \\ k_{12}=\frac{1}{\rho}\left(b_{2}+b_{I}\right)\left(\frac{b_{2}+b_{I}}{c_{2}}+\frac{a_{2}}{c_{2}}\frac{l}{\rho+\delta}\right)-\frac{c_{2}}{2}\frac{1}{\rho}\left(\frac{b_{2}+b_{I}}{c_{2}}+\frac{a_{2}}{c_{2}}\frac{l}{\rho+\delta}\right)^{2}-\frac{1}{\rho}C_{I}+\frac{1}{\rho}C_{O} \\ +\frac{l}{\rho+\delta}\frac{1}{\rho}a_{2}\left(\frac{b_{2}+b_{I}}{c_{2}}+\frac{a_{2}}{c_{2}}\frac{l}{\rho+\delta}\right) \end{matrix}\right.$ (68)

Therefore, it can be concluded that:

$\begin{matrix} V_{I1}^{*}=\frac{l}{\rho+\delta}x_{I1}+\frac{1}{\rho}\left(b_{1}+b_{I}\right)\left(\frac{b_{1}+b_{I}}{c_{1}}+\frac{a_{1}}{c_{1}}\frac{l}{\rho+\delta}\right)-\frac{c_{1}}{2}\frac{1}{\rho}\left(\frac{b_{1}+b_{I}}{c_{1}}+\frac{a_{1}}{c_{1}}\frac{l}{\rho+\delta}\right)^{2}-\frac{1}{\rho}C_{I}-\frac{1}{\rho}C_{O} \\ +\frac{\partial V_{I1}}{\partial x_{I1}}\frac{1}{\rho}a_{1}\left(\frac{b_{1}+b_{I}}{c_{1}}+\frac{a_{1}}{c_{1}}\frac{l}{\rho+\delta}\right) \end{matrix}$ (69)

$\begin{matrix} V_{I2}^{\text{*}}=\frac{l}{\rho+\delta}x_{I2}+\frac{1}{\rho}\left(b_{2}+b_{I}\right)\left(\frac{b_{2}+b_{I}}{c_{2}}+\frac{a_{2}}{c_{2}}\frac{l}{\rho+\delta}\right)-\frac{c_{2}}{2}\frac{1}{\rho}\left(\frac{b_{2}+b_{I}}{c_{2}}+\frac{a_{2}}{c_{2}}\frac{l}{\rho+\delta}\right)^{2}-\frac{1}{\rho}C_{I}+\frac{1}{\rho}C_{O} \\ +\frac{l}{\rho+\delta}\frac{1}{\rho}a_{2}\left(\frac{b_{2}+b_{I}}{c_{2}}+\frac{a_{2}}{c_{2}}\frac{l}{\rho+\delta}\right) \end{matrix}$ (70)

In this case,

$F_{I1}^{*}\left(t\right)=\frac{b_{1}+b_{I}}{c_{1}}+\frac{a_{1}}{c_{1}}\frac{l}{\rho+\delta}$ (71)

$F_{I2}^{*}\left(t\right)=\frac{b_{2}+b_{I}}{c_{2}}+\frac{a_{2}}{c_{2}}\frac{l}{\rho+\delta}$ (72)
